# Supplementary material for: Preventing and Treating Pain and Anxiety during Needle-Based Procedures in Children with Cancer in Low- and Middle-Income Countries
Source: Cancers (Basel). 2024 Mar 1;16(5):1025. doi: 10.3390/cancers16051025 (PMC10931017; doi:10.3390/cancers16051025)
Supplement: Supplementary file 1 [file cancers-16-01025-s001.zip › cancers-2861612-supplementary.pdf]

Supplemental Tables and Figures:

Preventing and Treating Pain and Anxiety during Needle-Based Procedures in Children with Cancer in Low- and Middle-Income Countries

Michael J. McNeil [1,2,*], Ximena Garcia Quintero [1], Miriam Gonzalez [1], Yawen Zheng [1], Cecilia Ugaz Olivares [3], Roxana Morales [3], Erica Boldrini [4], Débora Rebollo de Campos [4], Daiane Ferreira [4], Kamalina Coopasamy [5], Joliza Caneba [6], Maria Louisa Padernilla [6], Stefan Friedrichsdorf [7], Justin N. Baker [8] and Paola Friedrich [1]

Table of Contents

Item	Page
**Supplemental Figure S1:** Patient/Parent Satisfaction Card	2
**Supplemental Figure S2:** Provider Self Report Form	3
**Supplemental Figure S3:** Auditing Form	4
**Supplemental Table S1:** Outcome, Process, Balance Measures	6
**Supplemental Figure S4:** Patient/Parent Report of Pain During Procedure	6
**Supplemental Table S2:** Patient/Parent Satisfaction	7
**Supplemental Figure S5:** Patient Experience for Patient in Philippines	7
**Supplemental Figure S6A:** Self-Reported Percentage Use of Sucrose or Breastfeeding When Appropriate	7
**Supplemental Figure S6B:** Self-Reported Percentage Use of Topical Anesthesia	8
**Supplemental Table S3:** Number of Comfort Promise Interventions	8
**Supplemental Figure S7A:** Average needlestick procedure duration time	9
**Supplemental Figure S7B:** Average Attempts Per Patient Encounter	9
**Supplemental Table S4:** Provider Satisfaction	10

Supplemental Figure S1 Patient/Parent Satisfaction Card

Institutional Logo Here

The Comfort Promise

Date: MM / DD / YY
Time: ________ AM PM
Weekday: ☐
Weekend: ☐

1. What is your child's age? ______ (years) ______ (months)

2. Was your child in pain?

☐ Yes ☐ No

2. If Yes, do you think the hospital staff did everything they could to help prevent or alleviate the pain?

☐ Yes, definitely ☐ Yes, to some extent ☐ No

3. Where was the needle stick performed?

☐ Inpatient unit ☐ Outpatient clinic

4. Roughly, how many minutes did you have to wait for this needle-stick? __________________ minutes

Supplemental Figure S2 Provider Self-Report Form:

Institution Logo

The Comfort Promise

Facility code: Date/Time: MM/DD/YYYY (hh:mm)

Type of Unit: ☐ Inpatient ☐ Outpatient

Your role: ☐ Nurse ☐ Physician ☐ Phlebotomist

☐ Other (specify): ____________________

Patient Age: Child age _______ (years) ______(months)

Needle Stick Performed ☐ Lab Draw ☐ Peripheral IV Placement

How many attempts did it take? ____________________

Were age-appropriate positioning techniques used?	☐ Yes ☐ No
Were age-appropriate distraction techniques used?	☐ Yes ☐ No
Was a topical anesthetic used?	☐ Yes ☐ No
Was sucrose or breastfeeding used for infant less than 12 months?	☐ Yes ☐ No ☐ N/A

Start time for the procedure:	_______________
End time for the procedure:	_______________
Was the procedure successful?	☐ Yes ☐ No
How satisfied are you with how the procedure went?	☐ Very Satisfied ☐ Satisfied ☐ Somewhat Satisfied ☐ Not Satisfied

Supplemental Figure S3 Auditing Form

Institution
Logo

The Comfort Promise

Facility code:	Date/Time: MM/DD/YYYY (hh:mm)
Type of Unit: ☐ Inpatient ☐ Outpatient	Shift: ☐ Weekday ☐ Weekend
Performer role: ☐ Nurse ☐ Physician ☐ Phlebotomist ☐ Other (specify): ____________________	
Observer role: ☐ Nurse ☐ Physician ☐ Phlebotomist ☐ Other (specify): ____________________	

Availability

Were topical anesthetics readily available in the clinical area?	☐ Yes	☐ No
Were distraction supplies readily available in the clinical area?	☐ Yes	☐ No
Were reminders, posters, or educational materials available in the clinical area?	☐ Yes	☐ No

Needle-sticks Observations

Obs	Component	Obs	
1	Child age _______ (years) ______(months)<br>☐ age-appropriate distractions techniques<br>☐ age-appropriate positioning techniques<br>☐ topical anesthesia<br>☐ sucrose or breast feeding when appropriate<br>○ All components	2	Child age _______ (years) ______(months)<br>☐ age-appropriate distractions techniques<br>☐ age-appropriate positioning techniques<br>☐ topical anesthesia<br>☐ sucrose or breast feeding when appropriate<br>○ All components
3	Child age _______ (years) ______(months)<br>☐ age-appropriate distractions techniques<br>☐ age-appropriate positioning techniques<br>☐ topical anesthesia<br>☐ sucrose or breast feeding when appropriate<br>○ All components	4	Child age _______ (years) ______(months)<br>☐ age-appropriate distractions techniques<br>☐ age-appropriate positioning techniques<br>☐ topical anesthesia<br>☐ sucrose or breast feeding when appropriate<br>○ All components
5	Child age _______ (years) ______(months)<br>☐ age-appropriate distractions techniques<br>☐ age-appropriate positioning techniques<br>☐ topical anesthesia<br>☐ sucrose or breast feeding when appropriate<br>○ All components	6	Child age _______ (years) ______(months)<br>☐ age-appropriate distractions techniques<br>☐ age-appropriate positioning techniques<br>☐ topical anesthesia<br>☐ sucrose or breast feeding when appropriate<br>○ All components
7	Child age _______ (years) ______(months)<br>☐ age-appropriate distractions techniques<br>☐ age-appropriate positioning techniques<br>☐ topical anesthesia<br>☐ sucrose or breast feeding when appropriate<br>○ All components	8	Child age _______ (years) ______(months)<br>☐ age-appropriate distractions techniques<br>☐ age-appropriate positioning techniques<br>☐ topical anesthesia<br>☐ sucrose or breast feeding when appropriate<br>○ All components
9	Child age _______ (years) ______(months)<br>☐ age-appropriate distractions techniques<br>☐ age-appropriate positioning techniques<br>☐ topical anesthesia<br>☐ sucrose or breast feeding when appropriate<br>○ All components	10	Child age _______ (years) ______(months)<br>☐ age-appropriate distractions techniques<br>☐ age-appropriate positioning techniques<br>☐ topical anesthesia<br>☐ sucrose or breast feeding when appropriate<br>○ All components

Calculating Adherence			
**Component**	**Numerator[1]**	**Denominator[2]**	**Adherence[3]**
Age-appropriate distractions techniques			
Appropriate positioning techniques			
Topical anesthesia			
Sucrose or breast feeding when appropriate			
All components			

[1]Write the total number of providers who used each component during the needle-stick procedure.
[2] Write the total number of observations for that component; notice that some components are not applicable to all patients and depend on their age.
[3]Calculate by dividing the total of provider who used the component (numerator) by the total of observations(denominator) and multiply by 100.

Parents Satisfaction Survey Interviews

Int	Questions	Int	
1	Child age _______ (years) ______(months)<br>Parent reported pain ☐ Yes ☐ No<br>Hospital staff prevent or alleviate the pain:<br>☐ Yes, definitely ☐ Yes, to some extent ☐ No<br>Unit where needle stick was performed:<br>☐ Inpatient ☐ Outpatient<br>Needle-stick waiting time: _____________(minutes)	2	Child age _______ (years) ______(months)<br>Parent reported pain ☐ Yes ☐ No<br>Hospital staff prevent or alleviate the pain:<br>☐ Yes, definitely ☐ Yes, to some extent ☐ No<br>Unit where needle stick was performed:<br>☐ Inpatient ☐ Outpatient<br>Needle-stick waiting time: _____________(minutes)
3	Child age _______ (years) ______(months)<br>Parent reported pain ☐ Yes ☐ No<br>Hospital staff prevent or alleviate the pain:<br>☐ Yes, definitely ☐ Yes, to some extent ☐ No<br>Unit where needle stick was performed:<br>☐ Inpatient ☐ Outpatient<br>Needle-stick waiting time: _____________(minutes)	4	Child age _______ (years) ______(months)<br>Parent reported pain ☐ Yes ☐ No<br>Hospital staff prevent or alleviate the pain:<br>☐ Yes, definitely ☐ Yes, to some extent ☐ No<br>Unit where needle stick was performed:<br>☐ Inpatient ☐ Outpatient<br>Needle-stick waiting time: _____________(minutes)
5	Child age _______ (years) ______(months)<br>Parent reported pain ☐ Yes ☐ No<br>Hospital staff prevent or alleviate the pain:<br>☐ Yes, definitely ☐ Yes, to some extent ☐ No<br>Unit where needle stick was performed:<br>☐ Inpatient ☐ Outpatient<br>Needle-stick waiting time: _____________(minutes)	6	Child age _______ (years) ______(months)<br>Parent reported pain ☐ Yes ☐ No<br>Hospital staff prevent or alleviate the pain:<br>☐ Yes, definitely ☐ Yes, to some extent ☐ No<br>Unit where needle stick was performed:<br>☐ Inpatient ☐ Outpatient<br>Needle-stick waiting time: _____________(minutes)
7	Child age _______ (years) ______(months)<br>Parent reported pain ☐ Yes ☐ No<br>Hospital staff prevent or alleviate the pain:<br>☐ Yes, definitely ☐ Yes, to some extent ☐ No<br>Unit where needle stick was performed:<br>☐ Inpatient ☐ Outpatient<br>Needle-stick waiting time: _____________(minutes)	8	Child age _______ (years) ______(months)<br>Parent reported pain ☐ Yes ☐ No<br>Hospital staff prevent or alleviate the pain:<br>☐ Yes, definitely ☐ Yes, to some extent ☐ No<br>Unit where needle stick was performed:<br>☐ Inpatient ☐ Outpatient<br>Needle-stick waiting time: _____________(minutes)
9	Child age _______ (years) ______(months)<br>Parent reported pain ☐ Yes ☐ No<br>Hospital staff prevent or alleviate the pain:<br>☐ Yes, definitely ☐ Yes, to some extent ☐ No<br>Unit where needle stick was performed:<br>☐ Inpatient ☐ Outpatient<br>Needle-stick waiting time: _____________(minutes)	10	Child age _______ (years) ______(months)<br>Parent reported pain ☐ Yes ☐ No<br>Hospital staff prevent or alleviate the pain:<br>☐ Yes, definitely ☐ Yes, to some extent ☐ No<br>Unit where needle stick was performed:<br>☐ Inpatient ☐ Outpatient<br>Needle-stick waiting time: _____________(minutes)

Supplemental Table S1: Outcome, Process, and Balance Measures for Global Comfort Promise

Outcome Measures	Process Measures	Balance Measures
1. Realtime Patient/Parent satisfaction<br>2. Prevalence of Patient/Parent satisfaction	1. Frequency of use of the four Comfort Promise principles<br>2. *Utilization of ≥2 principles per patient encounter (calculated)*<br>3. Frequency of availability of topical anesthetics in clinical area<br>4. Frequency of availability of distraction supplies in clinical area	1. Length of time waiting for procedure to start<br>2. Length of time the procedure takes<br>3. Success rate of the procedure<br>4. Number of attempts per patient encounter<br>5. Provider satisfaction

Supplemental Figure S4: Patient/Parent Reporting Pain After Real-Time Needlestick Procedure

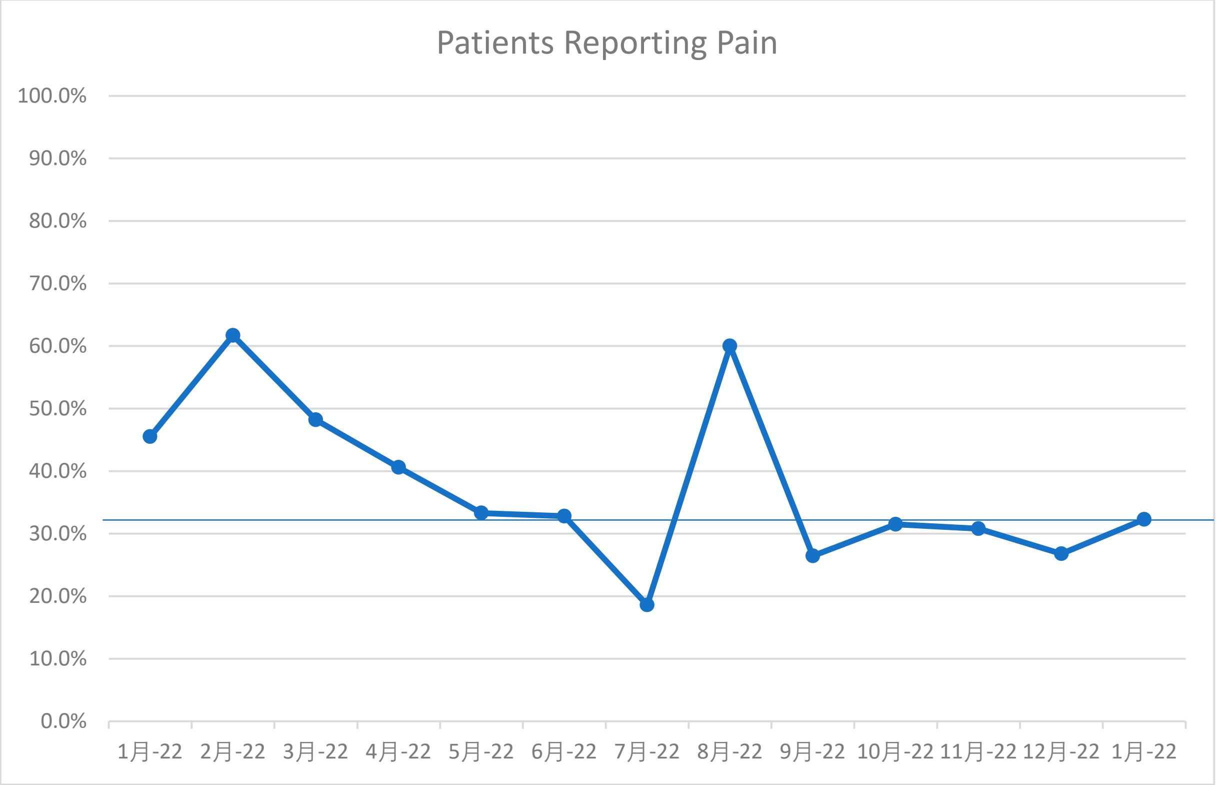

Supplemental Table S2: Patient/Parent Satisfaction

**Total**	**2199**
**Pain Y/N**	
Yes	743 (33.7%)
No	1456 (66.3%)
**If Yes, Was the Pain Alleviated Y/N**	
Yes, definitely	702 (94.7%)
Yes, to some extent	40 (5.3%)

Supplemental Figure S5:

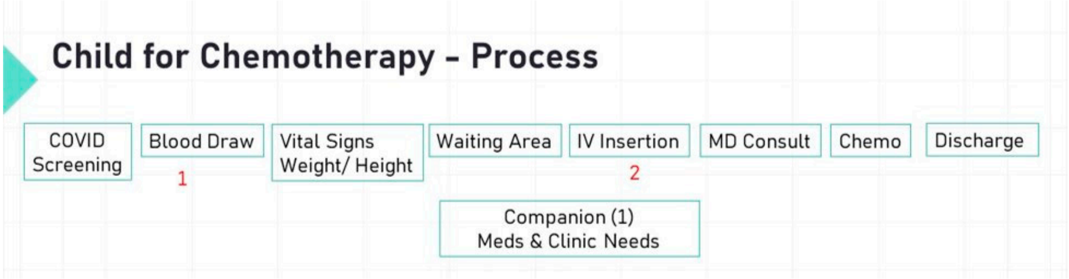

Supplemental Figure S6A: Self-Reported Percentage Use of Sucrose or Breastfeeding When Appropriate

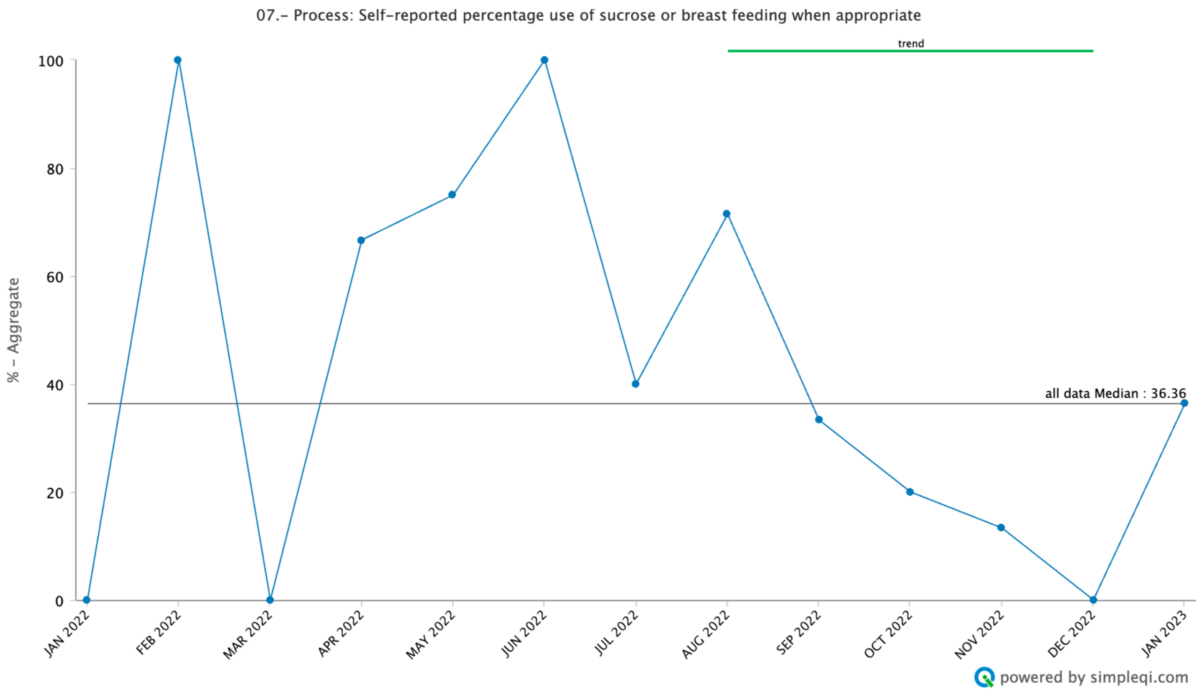

Supplemental Figure S6A: Percentage of healthcare professionals that recorded using sucrose or breastfeeding when appropriate during the needle-based procedure performed, over the total number of needle-based procedures performed. This data was collected each week and then aggregated into monthly data points for analysis.

Supplemental Figure S6B Self-Reported Percentage Use of Topical Anesthesia

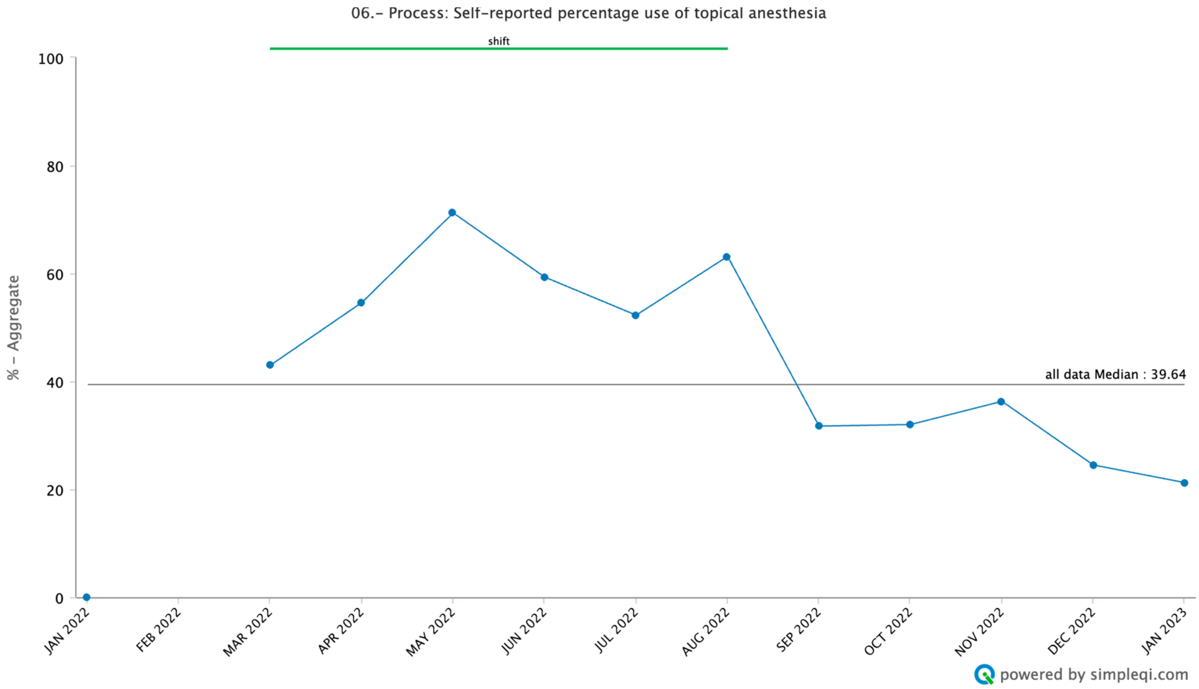

Supplemental Figure S6B: Percentage of healthcare professionals that recorded using anesthesia during the needle-based procedure performed, over the total number of needle-based procedures performed. This data was collected each week and then aggregated into monthly data points for analysis.

Supplemental Table S3: Number of Comfort Promise Interventions

#Comfort Promise Principles	N
1	15
2	1026
3	1075
4	69
TOTAL	2185

*Only children <2 had all 4 interventions.

Supplemental Figure S7A: Average needlestick procedure duration time

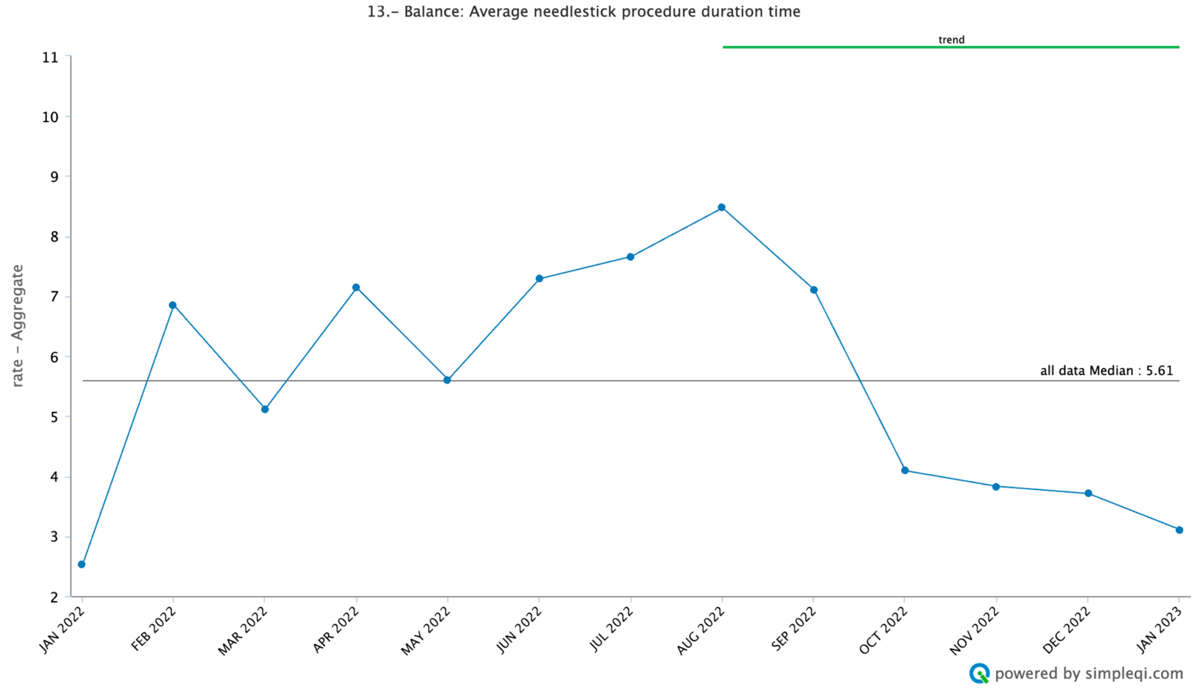

Supplemental Figure S7A: The time in minutes between procedure start and end time were recorded for each procedure. This was then recorded as a single number for each procedure. The average for each month was then obtained recorded in minutes.

Supplemental Figure S7B: Average Attempts Per Patient Encounter

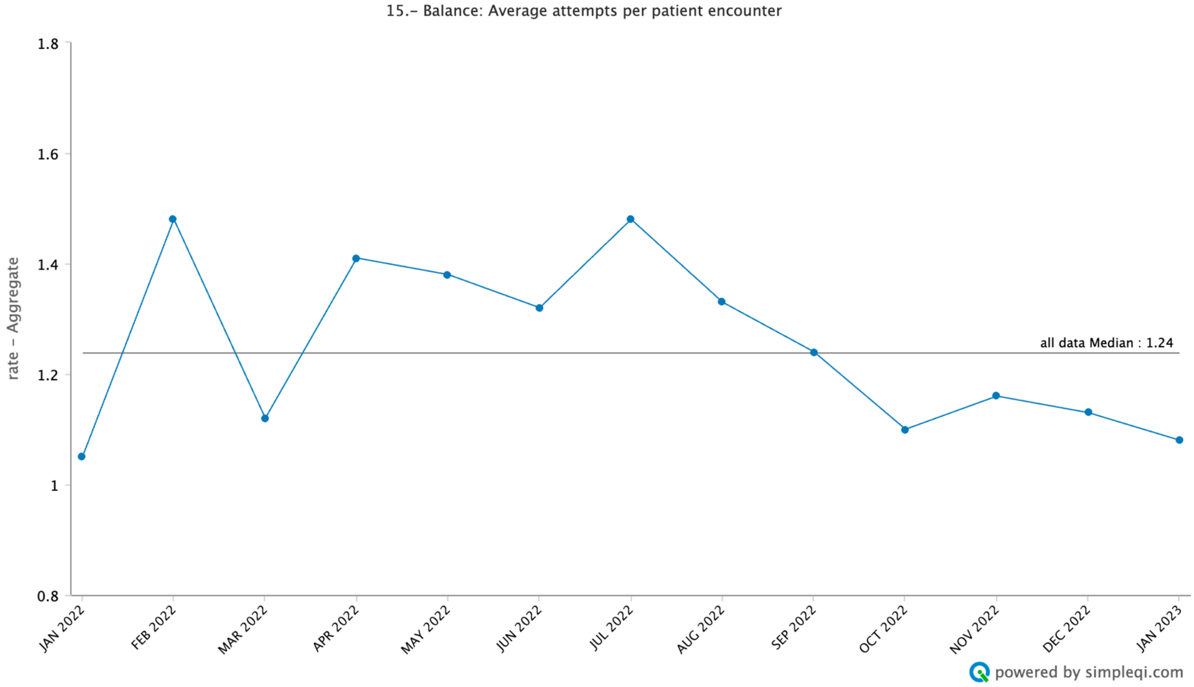

Supplemental Figure S7B: The number of attempts for the needlestick in minutes were recorded for each procedure. The average for each month was then obtained recorded.

Supplemental Table S4: Provider Satisfaction

Procedure Satisfaction	N	%
Very Satisfied	1632	74.60%
Satisfied	519	23.70%
Somewhat Satisfied	28	1.28%
Not Satisfied	6	0.27%
**TOTAL**	2185	99.85%
